# 149. Stewardship Efforts during the COVID-19 Pandemic: Impact on Antibiotic Prescribing Practices of Ambulatory Care Providers for Common (non-COVID-19) Viral Respiratory Syndromes

**DOI:** 10.1093/ofid/ofad500.222

**Published:** 2023-11-27

**Authors:** Melinda Mackey, Reese Cosimi, Florian Daragjati, Erin Rice, Collin Miller, Aaron Shoemaker, Mohamad G Fakih

**Affiliations:** Ascension Health, Evansville, Indiana; Ascension, Indianapolis, Indiana; Ascension, Indianapolis, Indiana; Ascension, Indianapolis, Indiana; Ascension Health, Evansville, Indiana; ascension, Nobelsville, Indiana; Ascension, Indianapolis, Indiana

## Abstract

**Background:**

An estimated one third of antibiotics prescribed in the outpatient setting is considered unnecessary. We evaluated ambulatory antimicrobial use for a single system before and during the COVID-19 pandemic and assessed the impact of a system-based stewardship intervention on overall antibiotic selection and prescribing rates for indications with typically viral etiologies.

Comparison of Antibiotic Use for Common Viral Respiratory Syndromes Prepandemic and Pandemic Periods

**Figure:**
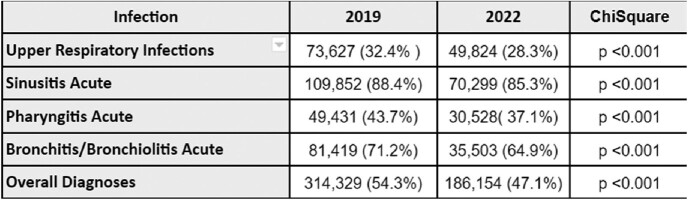

Change in Antibiotic Use and Spectrum for Prepandemic and Pandemic Periods

**Figure:**
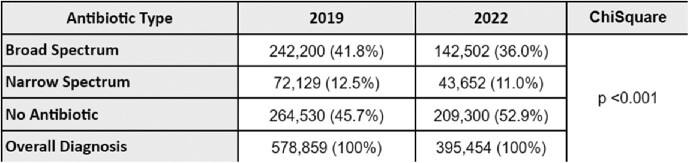

**Methods:**

This retrospective, quasi-experimental pre-post design evaluated rates of antibiotic prescribing for ambulatory setting in all ages across a large, multi-state healthcare system with encounters coded for common viral respiratory syndromes. Stewardship interventions were implemented starting July 2021 and included mandatory training on common upper respiratory infections for all Primary Care and Urgent Care clinicians, coupled with feedback on prescribing practices for targeted viral syndromes at quarterly intervals. Encounter volumes for ambulatory care clinicians were stratified by time period and common viral respiratory syndrome diagnosis (excluding COVID-19). Data on systemic antibiotic prescriptions collected from outpatient electronic health records (EHRs) was obtained during a prepandemic year (January 2019 - December 2019) and a pandemic year (January 2022 - December 2022). We compared the use of antibiotics for common viral respiratory syndromes between the two periods in the ambulatory setting and further evaluated for any changes in antibiotic selection.

**Results:**

Ambulatory antibiotic utilization for common viral respiratory syndromes decreased from 54.3% (prepandemic) to 47.1% (pandemic), resulting in an absolute 7.2% reduction (p< 0.001). After adjusting for the decreased volume of common viral syndromes diagnosed during 2022, approximately 28,578 fewer antibiotics were prescribed during the COVID period.

**Conclusion:**

Associated with an intervention to optimize antimicrobial use, we witnessed an improvement in overall use and choice of antimicrobials during the pandemic in a single system.

**Disclosures:**

**Melinda Mackey, MSN, RN, CPHQ, CCM**, Pfizer, Inc: Advisor/Consultant **Reese Cosimi, PharmD**, Allergen: Advisor/Consultant

